# R-Spondin 2 Induces Odontogenic Differentiation of Dental Pulp Stem/Progenitor Cells via Regulation of Wnt/β-Catenin Signaling

**DOI:** 10.3389/fphys.2020.00918

**Published:** 2020-08-07

**Authors:** Yuping Gong, Shuai Yuan, Jingjing Sun, Ying Wang, Sirui Liu, Runying Guo, Wenhang Dong, Rui Li

**Affiliations:** ^1^Department of Oral and Maxillofacial Surgery, The First Affiliated Hospital of Zhengzhou University, Zhengzhou, China; ^2^Department of Clinical Medicine, Academy of Medical Sciences at Zhengzhou University, Zhengzhou, China

**Keywords:** R-spondin 2, dental pulp stem/progenitor cells, odontoblast differentiation, Wnt/β-catenin signaling, DKK-1, Wnt3a

## Abstract

Odontoblast cells generated from human dental pulp stem/progenitor cells (hDPSCs) secrete reparative dentin in responds to an injury. Endogenous Wnt signaling is also activated during this process, and these Wnt-activated cells are responsible for the following repair response. R-spondin 2 (Rspo2) is a potent stem cell growth factor, which strongly potentiates Wnt/β-catenin signaling and plays a vital role in cell differentiation and regeneration. However, the role of Rspo2 during odontoblast differentiation in hDPSCs has not yet been completely understood. This study investigated the effects of Rspo2 on hDPSCs to provide therapeutic insight into dentin regeneration and reparative dentin formation. HDPSCs were extracted from human molars or premolars. Immunofluorescence staining and flow cytometric analysis were used to detect the mesenchymal stem cell markers in hDPSCs. EdU assay and Cell Counting Kit-8 (CCK-8) were performed to explore cell proliferation. The odontogenic differentiation levels were determined by measuring the mRNA and protein expression of DSPP, DMP-1, ALP, and BSP. Immunofluorescence staining was performed to detect the localization of β-catenin. The biological effects of Rspo2 on hDPSCs was investigated using the Lentivirus-based Rspo2 shRNA and recombined human Rspo2 (rhRspo2). Recombined human DKK-1 (rhDKK-1) and recombined human Wnt3a (rhWnt3a) were used for further investigation. The cells generated from human dental pulp expressed mesenchymal stem cell markers Vimentin, Stro-1, Nestin, C-kit, CD90, and CD73, while were negative for CD3, CD31, and CD34. The mRNA expression levels of the odontogenic-related genes DSPP, DMP-1, ALP, and BSP were upregulated in the rhRspo2 treated cells. Silencing Rspo2 suppressed the proliferation and differentiation of the hDPSCs. Blockade of Wnt signaling with DKK-1 inhibited Rspo2-induced activation of Wnt/β-catenin signaling and cell differentiation. The combined use of rhWnt3a and rhRspo2 created a synergistic effect to improve the activation of Wnt/β-catenin signaling. Rspo2 promoted the proliferation and odontogenic differentiation of hDPSCs by regulating the Wnt/β-catenin signaling pathway.

## Introduction

Several conditions can lead to exposure of the pulp of a tooth, such as dental caries, trauma, or mechanical responses. The consequences of pulp exposure may be severe, requiring a root canal treatment or extraction. An alternative therapy is direct pulp capping, by placing a material directly over the exposed pulp tissue, in an effort to retain pulpal vitality and health. A successful direct pulp capping can avoid the loss of a tooth or the need for replacement. Calcium hydroxide and trioxide mineral aggregates (MTA) are common pulp capping materials. Calcium hydroxide has consistently been considered the “gold standard” of the available direct pulp capping materials(Hilton, 2009; Hilton et al., 2013). However, this treatment has several limitations that hinder application, including high solubility in oral fluids, lack of adhesion qualities, and “tunnel defects” within the reparative dentin (Hilton, 2009). MTA is considered to be preferable to calcium hydroxide, as it often results in a more favorable outcome (Parolia et al., 2010). However, it can produce a color change effect (Aeinehchi et al., 2003) and exhibits high solubility (Fridland and Rosado, 2003), so this procedure is less desirable to the patient. Therefore, the development of a more effective direct pulp capping material is a long-term goal of dental pulp therapy.

Recombinant growth factors have been successfully used in clinical applications. For example, BMP-2 and BMP-7 have been approved by the FDA in the United States for spine fusion surgery (Even et al., 2012). Canonical Wnt signaling plays a vital role in tissue generation, regeneration, and self-renewal (Clevers et al., 2014). Growth factors involved in regulating the Wnt signaling pathway may potentially be promising molecules utilized to enhance dentin regeneration. Odontoblast cells generated from human dental pulp stem cells secrete reparative dentin to preserve the vitality and function of the existing teeth following injury (Gronthos et al., 2000; Gong et al., 2012; Babb et al., 2017). Endogenous Wnt signaling will be activated when the pulp responds to injury, and these Wnt-activated cells are responsible for the subsequent repair response (Zhao et al., 2018). Therefore, cytokines as pulp capping agents could show efficacy in the process of treating pulp exposure by activating the Wnt/β-catenin signaling pathway and promoting the formation of reparative dentin.

R-spondin proteins (Rspo1-4) are potent stem cell growth factors which strongly potentiate Wnt/β-catenin signaling (Kim et al., 2008; Glinka et al., 2011). Several studies have demonstrated that R-spondin proteins contribute to the regulation of tissue patterning and differentiation (Zhou et al., 2012; Storm et al., 2016; Zhu et al., 2016). Rspo2 has been reported to have the capacity of promoting osteoblast differentiation within MC3T3 E1 cells (Friedman et al., 2009; Zhu et al., 2016), mouse bone marrow stem cells (Knight et al., 2018), and human periodontal ligament cells (Arima et al., 2019) by modulating Wnt/β-catenin signaling. Bone marrow stem cells (BMSCs) isolated from Rspo2^Ftl^ mice (deficient in Rspo2) showed reduced osteogenesis compared with BMSCs from wild-type litter mates (Knight et al., 2018). Rspo2 is highly expressed by osteoblasts *in vitro* and sufficient for Wnt/β-catenin signaling activation and enhanced osteoblast maturation or mineralization (Friedman et al., 2009). However, the role of Rspo2 during odontoblast differentiation of the hDPSCs and dentin formation has not yet been thoroughly investigated. DPSCs and BMSCs share many features. The biochemical pathways involved in the differentiation between DPSCs and the functional odontoblast, and BMSCs into osteoblasts are similar (Gronthos et al., 2000). Therefore, we hypothesized that Rspo2 might be a candidate for use in odontogenic differentiation of hDPSCs and reparative dentin formation.

In the present study, we examined the effects of Rspo2 in the proliferation and odontoblast differentiation of hDPSCs *in vitro*. We also investigated the pathway involved. We hypothesized that Rspo2 should be an agonist of hDPSCs to promote odontogenic differentiation through the Wnt/β-catenin signaling pathway.

## Materials and Methods

### Cell Isolation and Culture

Human third molars or premolars were obtained from patients (18–25 years old) at The First Affiliated Hospital of Zhengzhou University. All of the donors signed an informed consent form. HDPSCs were isolated and cultivated, as described previously (Gronthos et al., 2000). Cells from passage 3 and 4 were used for the experiments and cultured in Dulbecco’s modified Eagle’s medium (DMEM; Hyclone, United States), which was supplemented with 10% fetal bovine serum (FBS; Hyclone, United States), and 100 U/mL penicillin, plus 100 μg/mL streptomycin from (Solarbio, China) at 37°C under 5% CO_2_, the medium was changed every 3 days.

Different concentrations, either 0, 50, 100, or 200 ng/mL, of recombinant human Rspo2 (rhRspo2) from PeproTech (United States) were added to the medium for use in subsequent experiments. In addition, 100 ng/mL recombinant human Wnt3a (rhWnt3a) from R&D Systems (United States) or 800 ng/mL recombinant human Dickkopf-related protein 1 (DKK-1; rhDKK-1) from PeproTech (United States) were used in this study.

### Odontogenic Differentiation

For odontogenic differentiation, hDPSCs were cultured with odontogenic inductive medium containing 10% FBS, 10 mM β-glycerophosphate, 50 mg/ml ascorbic acid, and 100 nM dexamethasone (Sigma-Aldrich, United States) for 10 days, the medium was changed every 3 days. Differentiated cells then were collected for the quantitative real-time PCR (qRT-PCR), immunofluorescence staining, and western blot analysis.

### Flow Cytometry

Flow cytometric analysis was used to detect the surface marker of hDPSCs. A total of 1 × 10^6^ cells were trypsinized and incubated with anti-human CD90, CD73, CD3, CD33, and CD34 antibodies, respectively. All of the antibodies were purchased from BD Biosciences (United States). Flow cytometric analysis was conducted on a FACSAria II flow cytometer (BD Biosciences, United States).

### Immunofluorescence Staining

A total of 5 × 10^4^ cells were seeded on coverslips in each well of 24-well plates. At 80% confluence, cells were fixed with 4% formaldehyde, followed by permeabilized treatment with 0.5% Triton X-100. Subsequently, the cells were blocked with 5% bovine serum albumin (BSA) and then incubated at 4°C overnight with the following primary antibodies: anti-Stro-1 (mouse anti-human; 1:100; R&D systems, United States), anti-Vimentin (rabbit anti-human; 1:500; Abcam, United States), anti-Nestin (mouse anti-human; 1:100; Santa Cruz, United States), anti-C-kit (rabbit anti-human; 1:400; Cell Signaling Technology, United States), anti-dentin sialophosphoprotein (DSPP) (mouse anti-human; 1:100; Santa Cruz, United States), anti-osteopontin (OPN) (rabbit anti-human; 1:100; Proteintech, China), anti-dentin matrix acidic phosphoprotein 1 (DMP-1) (mouse anti-human; 1:100; Santa Cruz, United States), anti- alkaline phosphatase (ALP) (rabbit-anti-human; 1:100; Abcam, United States), anti-osteocalcin (OCN) (mouse anti-human; 1:200; Abcam, United States), anti-bone sialoprotein (BSP) (rabbit anti-human; 1:100; Abcam, United States) and anti-active β-catenin (rabbit anti-human; 1:800; Cell Signaling Technology, United States). The cells were washed and incubated with fluorescent-labeled secondary antibodies Dylight 488 goat anti-mouse IgG or Dylight 594 goat anti-rabbit IgG (1:250; Abbkine, United States) for 1 h at room temperature. The coverslips were moved and mounted on glass slides with the presence of 10 mg/mL DAPI (4′6-diamidino-2-phenylindole) (Solarbio, China) for nuclei staining. Cell images were recorded using a fluorescence microscope (Olympus, United States) or a ZEISS confocal microscope (Germany).

For DSPP, OPN, DMP1, ALP, OCN, and BSP detection, cells were incubated in odontogenic differentiation medium with 0, 50, or 200 ng/mL rhRspo2 for 10 days before fixation. For β-catenin detection, cells were stimulated with or without rhRspo2 (200 ng/mL), rhDKK-1 (800 ng/mL), or rhWnt3a (100 ng/mL) for 2 h before fixation.

### EdU Assay

A total of 1 × 10^4^ hDPSCs were seeded into 96-well plates in complete medium (DMEM supplemented with 10% FBS). The next day, the medium was removed, and the cells were incubated in DMEM without FBS for 24 h. Next, the medium was changed to complete medium with different concentrations of rhRspo2 (0, 50, 100, or 200 ng/mL). After 24 h, the proliferation of hDPSCs was assessed using the EdU assay (Ribobio, China). Following incubation, the medium was removed, and the cells were incubated in DMEM containing 50 μM EdU for 2 h at 37°C. The cells were fixed with 4% formaldehyde, and were then incubated in the dark with Apollo dye solution for 30 min followed by a Hoechst33343 reaction solution for 30 min in room temperature. Using the fluorescence microscope (Olympus Microscopes, United States), images were recorded.

### Cell Counting Kit-8 (CCK-8)

A total of 5 × 10^3^ hDPSCs were seeded into 96 well plates in complete medium. The next day, different concentrations of rhRspo2 (0, 10, 50, 100, or 200 ng/mL) were added to the medium. Following 0, 1, 2, 3, 4, and 5 days of incubation, the proliferation of hDPSCs was assessed using the CCK-8 assay (Dojindo, Japan). Briefly, following incubation, the medium was removed, and the cells were incubated in DMEM containing CCK-8 for 1 h at 37°C. The optical density (OD) of each well was read at 450 nm using a microplate reader CMax Plus (Molecular Devices, United States).

### Lentivirus Transfection

Lentivirus-based Rspo2 shRNA (shRspo2) and non-targeted negative control shRNA (shNC) were constructed, respectively (Hanheng, China). Approximately 0.6–1 × 10^5^ cells were seeded into a 6-well plate. Infection was performed using the lentivirus at a MOI (multiplicity of infection) of 40 when cells were grown to a confluence of 30%. After 12–16 h, the medium was replaced with fresh complete medium. In addition, 72 h after infection, the frequency of the green fluorescent protein (GFP)-positive cells were used to assess the efficiency of infection. Thereafter, the cells were treated with 3 μg/mL puromycin for 5 days to obtain stabled-transfected cells. The knockdown efficiency of Rspo2 was verified by quantitative real-time PCR and western blotting.

### Quantitative Real-Time PCR (qRT-PCR)

Total RNA was extracted from the cells with RNAiso Plus (TaKaRa, Japan) according to the manufacturer’s procedure. A 1 μg sample of total RNA was reverse-transcribed to cDNA utilizing the PrimeScript^TM^RT reagent Kit (TaKaRa, Japan). Thereafter, the cDNA sample was analyzed using TB Green^TM^Premix Ex Taq^TM^II (TaKaRa, Japan) on Quantstudio 5 Real-Time PCR system (Thermo Fisher Scientific, United States). Cycle conditions were as follows: 95°C for 30 s, followed by 45 cycles of 95°C for 5 s, 55°C for 30 s, and 72°C for 30 s. Primers are listed in Table 1. The data were analyzed using the ΔΔCT method.

**TABLE 1:** Primers used for the analysis of mRNA levels by qRT-PCR.

**Gene**	**Forward primer**	**Reverse primer**
DSPP	5′-CTGTTGGGAAGAGCCAAGATAAG-3′	5′-CCAAGATCATTCCATGTTGTCCT-3′
DMP-1	5′-GTGAGTGAGTCCAGGGGAGATAA3’	5′-TTTTGAGTGGGAGAGTGTGTGC-3′
ALP	5′-TAAGGACATCGCCTACCAGCTC-3′	5′-TCTTCCAGGTGTCAACGAGGT-3′
BSP	5′-GATTTCCAGTTCAGGGCAGTAG-3′	5′-CCCAGTGTTGTACAGAAAGTG-3′
GAPDH	5′-CTTTGGTATCGTGGAAGGACTC-3′	5′-GTAGAGGCAGGGATGATGTTCT-3′
LGR4	5′-CGGGCAACGACCTTTCTTT-3′	5′-CACAGATGCCGTAACTGAACAA-3′
LGR5	5′-TCCCTGCGTCTGGATGCTAA-3′	5′-CCAGGGAGTGGATTCTATTGTTATG-3′
LGR6	5′-TGCCTTATGCCTACCAGTGCT-3′	5′-CAGGTCCTGGTCATAGTGGTTCT-3′
ZNRF3	5′-CCAGAATTGGACCCGAAACC-3′	5′-TCGAGCCACTTTCTGCTTGTTG-3′
LRP5	5′-TGCTCCCACATCTGTATTGCC-3′	5′-CTCCTCGTCGCTCTGGTCAT-3′
LRP6	5′-CTTGCAGCCTGTGGGACTTAC-3′	5′-ATGTGAACAGCCACCATTATCC-3′
Frizzled4	5′-TCCCACCACAGAACGACCAC-3′	5′-AAGCCAGCATCATAGCCACACT-3′

### Western Blot Analysis

Cells were washed and lysed with RIPA lysis buffer (Beyotime, China) supplemented with 1 mM protease inhibitor and incubated on ice for 30 min. Subsequently, the lysate was centrifuged at 12,000 × g for 5 min at 4°C, and then the supernatant was collected. Proteins were separated by sodium dodecyl sulfate-polyacrylamide gel electrophoresis (SDS-PAGE) and transferred to polyvinylidene difluoride membranes (Millipore, United States). The membranes were blocked with 5% BSA in TBST and then incubated overnight at 4°C with the following primary antibodies: DSPP (rabbit anti-human; 1:1000; Bioworld, China), DMP-1 (mouse anti-human; 1:1000; Santa Cruz, United States), ALP (mouse anti-human; 1:500; Santa Cruz, United States), BSP (mouse anti-human; 1:500; Santa Cruz, United States), Non-phospho (Active) β-catenin (rabbit anti-human; 1:1000; Cell Signaling Technology, United States), β-catenin (rabbit anti-human; 1:1000; Cell Signaling Technology, United States) and GAPDH (mouse anti-human; 1:5000; Zen-Bio, China). HRP-conjugated goat anti-rabbit and goat anti-mouse secondary antibodies (1:10,000; EarthOx, United States) were used and then incubated with ECL plus (Beyotime, China) and visualized using an Amersham Imager 600 Super Sensitive Multifunctional Imaging Instrument (GE Healthcare, United States). The bands intensity were analyzed using Image J software.

### Statistical Analysis

All experiments were performed in triplicate and repeated three times. The data were analyzed using Prism6 software (Graphpad, United States), and statistical analyses were performed with the Student’s *t*-test. A *p* < 0.05 was considered statistically significant.

## Results

### Characterization of hDPSCs

**FIGURE 1:**
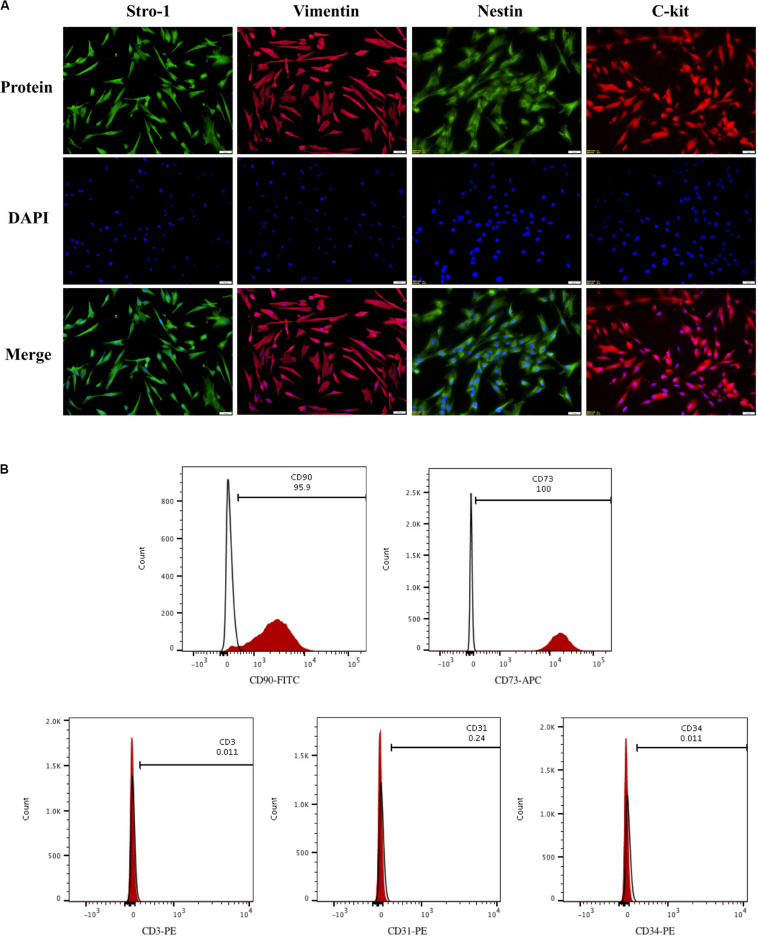
Identification of hDPSCs. HDPSCs were positive for Vimentin, Stro-1, Nestin, and C-kit **(A)**. Flow cytometric analyses revealed that hDPSCs expressed mesenchymal stem cell markers CD90 and CD73 and were negative for T Cell marker CD3, vascular endothelial cell marker CD31, and hematopoietic cell marker CD34 **(B)**. The experiments were performed for three times.

To evaluate hDPSCs, we examined these cells for the presence of mesenchymal stem cell markers by immunofluorescence and flow cytometry. As demonstrated by immunofluorescence, hDPSCs were positive for the mesenchymal stem cell markers, which include Vimentin, Stro-1, Nestin, and C-kit (Figure 1A). Results from the flow cytometry analysis showed that hDPSCs expressed mesenchymal stem cell markers, CD90 (95.9%) and CD73 (100%). However, the hDPSCs were negative for a T Cell marker, CD3 (0.011%), a vascular endothelial cell marker, CD31 (0.24%), and a hematopoietic cell marker, CD34 (0.011%) (Figure 1B).

### Effect of Rspo2 on the Proliferation and Differentiation of hDPSCs

**FIGURE 2:**
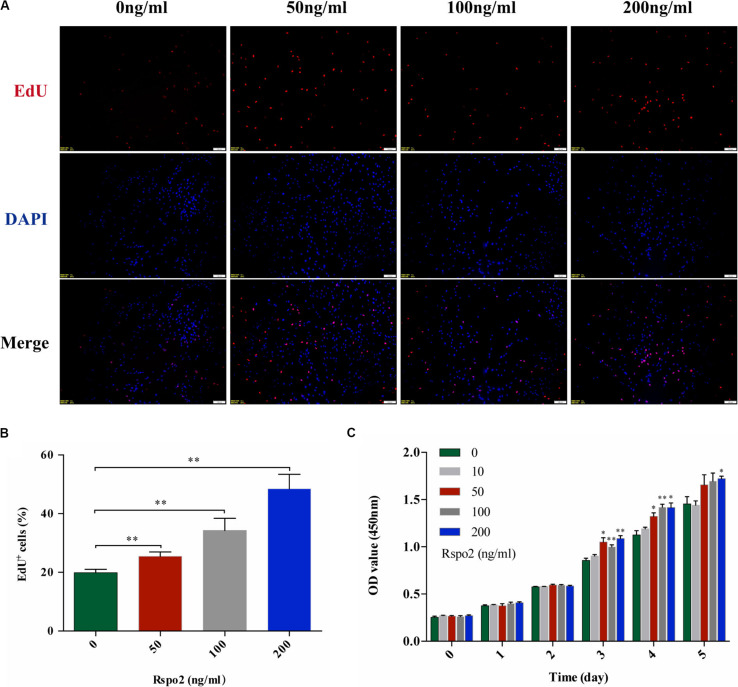
Effect of Rspo2 on the proliferation of hDPSCs. EdU **(A,B)** and CCK-8 assay **(C)** indicated enhanced proliferation of hDPSCs when the dose of Rspo2 is over 50 ng/mL. *P < 0.05 and **P < 0.01.

EdU and CCK-8 assays were performed to assess whether different concentrations of rhRspo2 could affect the proliferation of hDPSCs. As shown in Figures 2A,B, when the concentration of rhRspo2 is over 50 ng/mL, the proportion of cells a in proliferative stage were significantly higher compared with the 0 ng/mL condition. As demonstrated in Figure 2C, the optical density (OD) values began to have varying degrees of elevation when treated with rhRspo2 treatment group compared with the 0 ng/mL treatment group on the third day.

**FIGURE 3:**
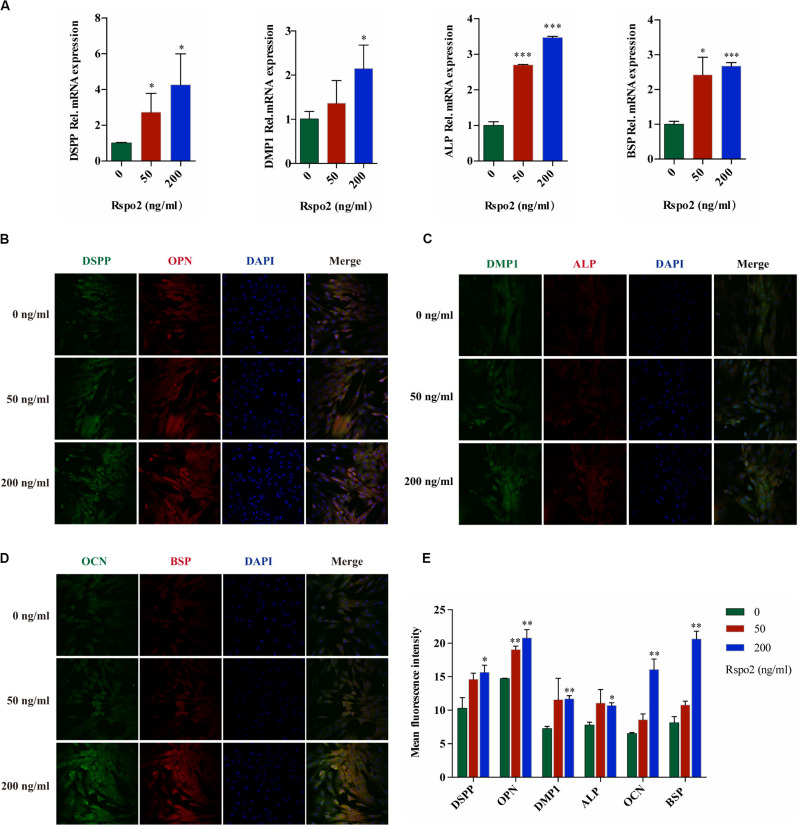
Effect of Rspo2 on the differentiation of hDPSCs. The expression of odontoblast-specific mRNA and protein in response to the exogenous rhRspo2 was assessed by qRT-PCR **(A)** and immunofluorescence staining **(B–E)**. *p < 0.05, **p < 0.01, and ***P < 0.001 compared with the control group.

In order to investigate the effect of Rspo2 on odontoblastic differentiation, cells were seeded into a 6-well plate and treated with odontogenic inductive medium with either 0, 50, and 200 ng/mL rhRspo2 for 10 days. We performed qRT-PCR to examine the gene expression of the odontogenic-related markers, which include DSPP, DMP-1, ALP, and BSP. The mRNA expression levels of these genes were up-regulated in rhRspo2-treated cells, with the highest mRNA expression levels found with the 200 ng/mL treatment group (Figure 3A).

In addition, using fluorescent microscopy to co-stain the markers DSPP, OPN, DMP-1, ALP, OCN, and BSP, we measured changes in fluorescent intensity as a readout to assess the function of odontogenic differentiation in hDPSCs. The fluorescence intensity increased significantly when the concentration of rhRspo2 was 200 ng/mL (Figures 3B–E).

### Silence of Rspo2 Suppressed the Proliferation and Differentiation of hDPSCs

**FIGURE 4:**
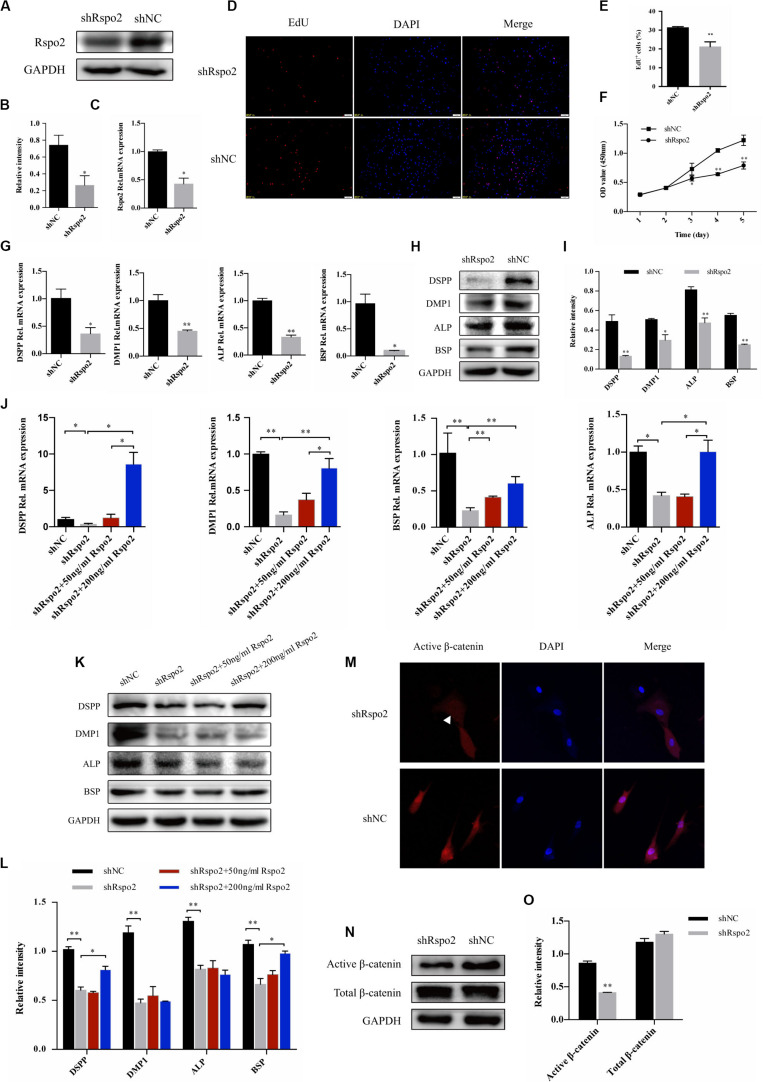
Down-regulation of proliferation, odontogenic differentiation and β-catenin in response to the knockdown of Rspo2. The knockdown efficiency of Rspo2 was verified by qRT-PCR and western blotting **(A–C)**. EdU **(D,E)** and CCK-8 assay **(F)** indicated a reduced proliferation of shRspo2 cells compared with the control. The mRNA and protein expression of DSPP, DMP-1, ALP, and BSP of shRspo2 and shNC cells following 10 days of odontogenic induction **(G–I)**. A rescue experiment was performed by adding 50 or 200 ng/mL rhRspo2 to treat shRspo2 cells **(J–L)**. The expression and localization of the β-catenin of shRspo2 cells and shNC cells was detected by immunofluorescence **(M)** and western blotting **(N,O)**. The experiments were performed for three times. *p < 0.05, and **p < 0.01 compared with the control group.

To further explore the effect of Rspo2 on hDPSCs, we successfully constructed Rspo2 knockdown cells using Lentivirus-based Rspo2 shRNA. The knockdown efficiency of Rspo2 was verified using qRT-PCR and western blotting (Figures 4A–C). Using the EdU and CCK-8 assays, we analyzed the proliferation ability of hDPSCs. Compared with shNC cells, Rspo2 knockdown significantly downregulated hDPSC proliferation (Figures 4D–F).

Cells were seeded into 6-well plates and treated with an odontogenic medium to confirm whether silencing Rspo2 would influence odontogenic differentiation in hDPSCs. After 10 days, cell lysates were collected, and the mRNA and protein levels of the odontoblast markers, DSPP, DMP1, ALP, and BSP, were examined. We found that silencing Rspo2 impaired the odontogenic potential of hDPSCs significantly (Figures 4G–I). We also performed a rescue experiment by adding exogenous rhRspo2 to the odontogenic culturing medium in shRspo2 cells. At concentrations of 200 ng/mL, rhRspo2 inhibited the downregulation of the odontoblast markers in the Rspo2 knockdown cells (Figures 4J–L). However, a higher concentration of rhRspo2 may be needed to rescue the protein expression of DMP-1 and ALP.

### Rspo2 Promoted Odontogenic Differentiation of hDPSCs by Regulating the Wnt/β-Catenin Signaling Pathway

Wnt signaling pathway played a pivotal role in odontoblast differentiation (Chen et al., 2009). Previous studies have confirmed that Rspo2 was capable of activating canonical Wnt/β-catenin signaling and facilitated the differentiation of stem cells (Arima et al., 2019). We hypothesized that the silence of Rspo2 produced an effect on Wnt signaling, and then inhibited the differentiation of hDPSCs. We measured the protein expression of β-catenin in the shRspo2 cells and shNC cells. Immunofluorescence assay confirmed a reduction in nucleus active β-catenin in the shRspo2 cells (Figure 4M). Western blot analysis demonstrated that the protein level of active β-catenin was significantly lower in the shRspo2 cells than in shNC cells (Figures 4N,O).

**FIGURE 5:**
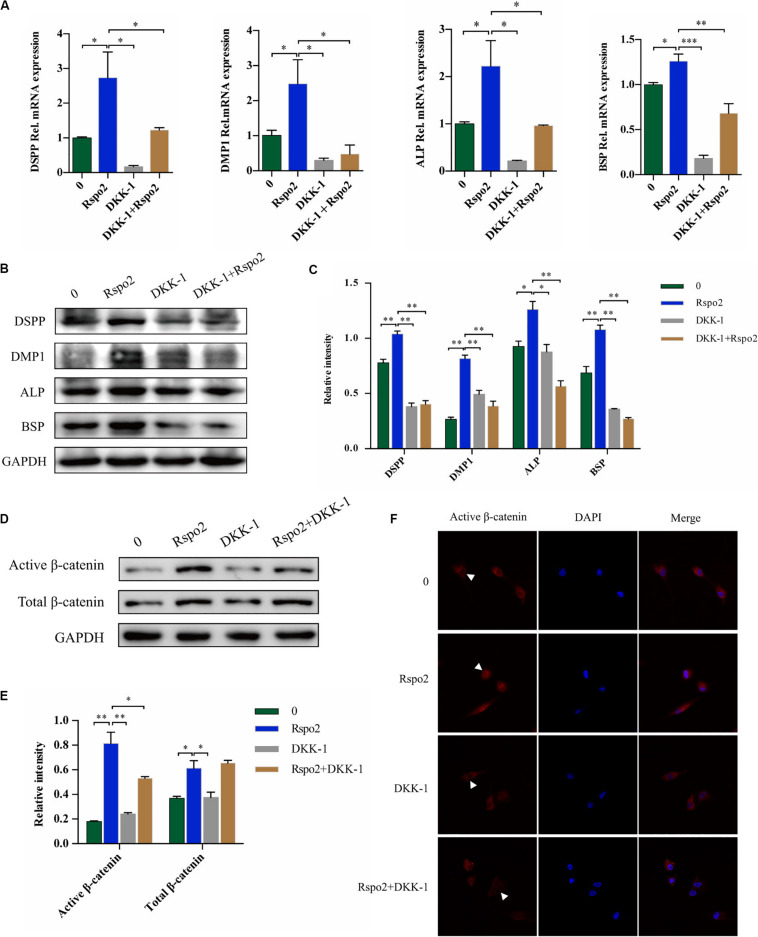
Blockade of Wnt signaling with DKK-1 can inhibit Rspo2-induced activation of Wnt/β-catenin signaling and cell differentiation. The mRNA and protein expression of DSPP, DMP-1, ALP, and BSP of cells treated with or without rhRspo2 (200 ng/mL) or DKK-1 (800 ng/mL) after 10 days of odontogenic induction **(A–C)**. The expression **(D,E)** and localization **(F)** of β-catenin in cells treated with or without rhRspo2 (200 ng/mL) or DKK-1 (800 ng/mL) for 2 h. The experiments were performed for three times. *p < 0.05, **p < 0.01, and ***p < 0.001 compared with the control group.

Dickkopf-related protein 1 (DKK-1) is a ligand for the Wnt receptors LRP5 and LRP6 and a negative regulator of Wnt/β-catenin signaling (Ren et al., 2013). Therefore, we utilized rhDKK-1 to further validate the association between Rspo2-mediated odontoblast differentiation and the Wnt/β-catenin signaling pathway. We used odontogenic medium to induce the cells by treating with or without the rhRspo2 (200 ng/mL) or the rhDKK-1 (800 ng/mL). We changed the culture medium every 3 days and added fresh recombinant human protein each time. After 10 days, the odontogenic differentiation markers in the hDPSCs were evaluated. The mRNA and protein levels of DSPP, DMP-1, ALP, and BSP were all significantly lower in the cells treated with both rhRspo2 and rhDKK-1 compared with levels in cells treated with rhRspo2 alone (Figures 5A–C).

In addition, the expression level and localization of β-catenin were assessed by western blotting and immunofluorescence, respectively. We induced the cells by treating with or without the rhRspo2 (200 ng/mL) or the rhDKK-1 (800 ng/mL) for 2 h. As depicted in Figures 5D,E, protein expression levels of active β-catenin were increased in the cells treated with rhRspo2, while significantly attenuated with in cells treated with rhDKK-1. Further, the levels of active β-catenin in nucleus was significantly upregulated in cells treated with rhRspo2, whereas the level was downregulated in cells treated with DKK-1 (Figure 5F).

### Wnt3a and Rspo2 Had a Synergistic Effect on Wnt/β-Catenin Signaling Activation of hDPSCs

**FIGURE 6:**
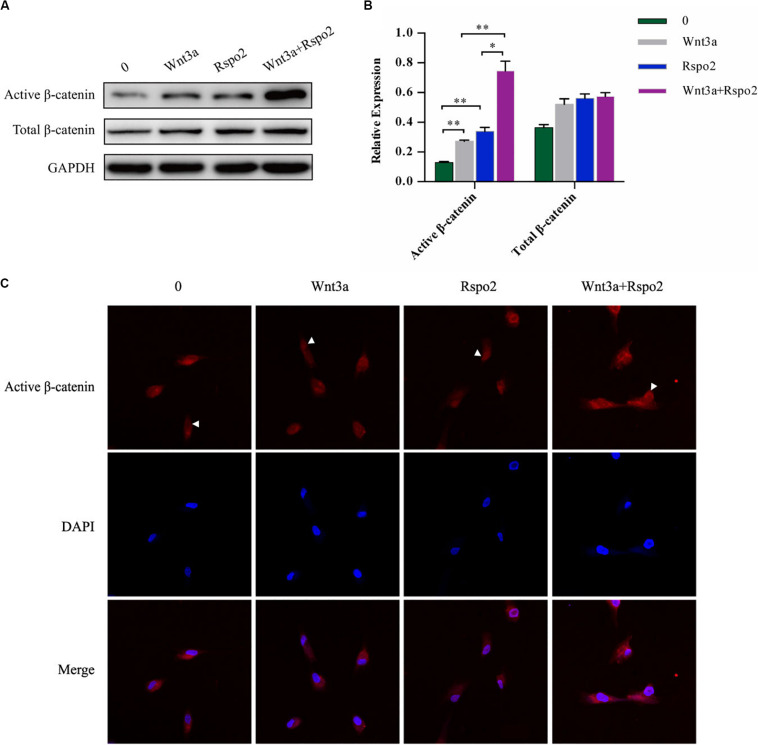
Wnt3a and Rspo2 produced a synergistic effect on Wnt/β-catenin signaling activation of the hDPSCs. The expression **(A,B)** and localization **(C)** of β-catenin in cells treated with or without rhRspo2 (200 ng/mL) or rhWnt3a (100 ng/mL) for 2 h. The experiments were performed for three times. *p < 0.05 and **p < 0.01 compared with the control group.

Wnt3a is a Wnt protein that could activate the canonical β-catenin pathway and induces Tcf/LEF transcription factors to regulate the expression of the target genes (Zhang et al., 2015). ZNRF3 could turn over the Wnt3a receptors frizzled and LRP6; however, this activity was inhibited by Rspo2 (Hao et al., 2012). Therefore, we hypothesized that Rspo2 and Wnt3a may have a synergistic effect to activate the Wnt/β-catenin pathway in hDPSCs. We induced the cells by treating with or without the rhWnt3a (100 ng/mL) or the rhRspo2 (200 ng/mL). After 2 h, the protein expression level of the active β-catenin was significantly higher in cells treated with both rhWnt3a and rhRspo2 compared with cells treated with either rhWnt3a or rhRspo2 alone (Figures 6A,B). Furthermore, the levels active β-catenin present is the nucleus was significantly upregulated in cells treated with rhWnt3a and rhRspo2 (Figure 6C).

### LGR4 May Be Involved in the Activation of Wnt/β-Catenin Signaling by Rspo2 in hDPSCs

**FIGURE 7:**
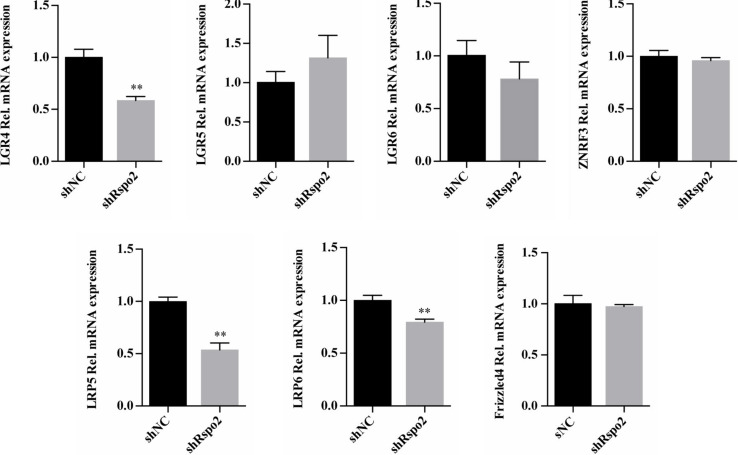
The mRNA expression of Wnt and Rspo2 related receptors. The experiments were performed for three times. ^∗∗^*p* < 0.01 compared with the control group.

We compared the mRNA expression levels of Wnt and Rspo2 related receptors between shRspo2 cells and shNC cells. As depicted in Figure 7, the expression of Wnt related receptors LRP5, LRP6, and Rspo2 related receptor LGR4 significantly decreased in shRspo2 cells. However, the expression of Wnt related receptors Frizzled4 and Rspo2 related receptors LGR5, LGR6, and ZNRF3 did not have significant change.

## Discussion

Stem cells isolated from different tissues such as bone marrow, muscle, adipose tissue, etc. all share two basic stem cell characteristics including self-renewal and the ability of multilineage differentiation. Stem cells derived from human dental pulp have been proven to have the advantages in myogenic differentiation, odontogenic/osteogenic differentiation, and chondrogenic differentiation (Zhang et al., 2006). HDPSCs are easily accessible, and have adequate sources, and have been considered for clinical use by many investigators. For example, hDPSCs have an exceptional differentiation capacity into osteoangiogenesis, which represent the gold standard for obtaining well vascularized bone (Paino et al., 2017).

Canonical Wnt signaling plays a vital role in tissue generation, regeneration, and self-renewal (Clevers et al., 2014). However, the function of Wnt/β-catenin signaling on odontoblast-like differentiation of hDPSCs has been controversial. Scheller demonstrated that canonical Wnt signaling inhibits the odontogenic differentiation of the hDPSCs (Scheller et al., 2008). Wnt10a, which is one of the Wnt agonists, has been reported to down-regulate the expression of odontoblast-specific genes in hDPSCs (Zhang et al., 2014). Other investigators have reported that β-catenin accumulation by various agonists promotes odontoblastic differentiation in hDPSCs (Kim et al., 2011, 2013; Han et al., 2014; Yoshida et al., 2016; Rahman et al., 2018). Therefore, it seems that different molecules that regulate Wnt/β-catenin signaling may lead to totally different outcomes. R-spondin are secreted proteins that function as stem cell growth factors (Sato et al., 2011), and have been demonstrated to strongly potentiate Wnt signaling, which includes both the canonical Wnt/β-catenin pathway and the non-canonical Wnt/PCP pathway (Hao et al., 2012). To have a deeper understanding of the canonical Wnt signaling pathway on odontoblast differentiation in hDPSCs and to provide more evidence for both dentin regeneration and reparative dentin formation, we sought to investigate the influence of R-spondin in hDPSCs.

Rspo2 has been reported to play a vital role in several types of differentiation, including neuroretina differentiation (Takata et al., 2017), chondrogenic differentiation (Takegami et al., 2016), and osteoblastic differentiation (Arima et al., 2019). To the best of our knowledge, our study is the first to report the impact of Rspo2 on odontoblast differentiation. We found that inducing odontogenic differentiation in combination with exogenously added Rspo2 in hDPSCs increased both mRNA and protein expression levels of DSPP, DMP1, ALP, and BSP, and the protein expression levels of OPN and OCN, whereas silencing Rspo2 significantly decreased the expression levels of these odontogenic markers. Rescue experiments indicated that exogenous Rspo2 could reverse the effect of Rspo2 depletion on odontogenic differentiation. All of these findings demonstrate that Rspo2 plays a promotive role during odontogenic differentiation in hDPSCs.

We found that inhibition of Wnt/β-catenin signaling by DKK-1 significantly suppressed the enhanced odontoblast differentiation seen with Rspo2 treatment alone. Further, we found that silencing Rspo2 significantly inhibited the expression of active β-catenin. These data suggest that Rspo2 produced a positive effect on the odontogenic differentiation in hDPSCs via modulating Wnt/β-catenin signaling. These data correspond to findings regarding the osteoblast induction ability of Rspo2.

The mechanism of activation for the Wnt/β-catenin signaling pathway by the R-spondin family is not fully understood. R-spondin functions via forming a ternary complex that prevents Wnt receptor degradation, which is mediated by RNF43 or ZNRF3 by binding to the R-spondin cognate receptors LGR4-6 and the E3 ubiquitin ligases RNF43 and ZNRF3 (Zebisch et al., 2013). It seems that R-spondins amplify the canonical Wnt pathway rather than activate it, while intestinal stem cells, which are required by both R-spondins and Wnt ligands, remained primed to differentiate (Huels and Sansom, 2017). In other cases, Rspo2 could inhibit RNF43 and ZNRF3 independently without binding to the LGR4-6 (Szenker-Ravi et al., 2018). Our study provides a molecular mechanism for potent odontogenic induction mediated through Rspo2. We found that Rspo2 could activate the Wnt signaling pathway, and combining Rspo2 and Wnt3a significantly amplified this pathway as shown by the expression of active β-catenin. Therefore, we conclude that Rspo2 is indispensable for the activation of Wnt/β-catenin signaling, and works synergistically with Wnt3a to amplify this pathway.

We also compared the mRNA expression levels of Wnt and Rspo2 related receptors between shRspo2 cells and shNC cells. Rspo2 may regulate Wnt/β-catenin signaling pathway through binding to LGR4 receptor. The lower expression levels of LRP5 and LRP6 in shRspo2 cells may lead to the downregulation of the Wnt/β-catenin signaling pathway activated through Wnt ligands. However, in order to clarify the real condition, further investigations are needed to determine whether these membrane receptors are involved in the remodeling events of Rspo2 during odontoblast differentiation.

Several studies have attempted to use growth factors or molecules for reparative dentin formation, such as Semaphorin 3A (Yoshida et al., 2016), Wnt3a (Hunter et al., 2015; Zhao et al., 2018), lithium chloride (Ishimoto et al., 2015), DMP-1 (Almushayt et al., 2006), and TGFβ-1 (Hu et al., 1998). Interestingly, most of these growth factors functioned by activating the Wnt/β-catenin signaling. We clarified that Wnt3a and Rspo2 create a synergistic effect on the enhancement of Wnt/β-catenin signaling. Several studies have demonstrated that Wnt3a plays a vital role in odontogenic differentiation (Sakisaka et al., 2015; Zhang et al., 2015; Rahman et al., 2018; Zhao et al., 2018). The combined use of Wnt3a and Rspo2 might be an alternative approach for odontogenic differentiation and reparative dentin formation.

## Conclusion

In summary, our findings suggested that Rspo2 played a vital role in the proliferation and odontogenic differentiation in hDPSCs, and knockdown of Rspo2 suppressed this activity. Inhibiting Wnt/β-catenin signaling by DKK-1 reduced the odontogenic effect of Rspo2. Wnt3a in combination with Rspo2 produced a synergistic effect during the activation of the Wnt/β-catenin signaling pathway. Our results demonstrated the feasibility of Rspo2 as a pulp capping agent to treat pulp exposure by activating endogenous Wnt signaling. Future work will focus on *in vivo* experiments to investigate the clinical uses of R-spondins and Wnt ligands, as well as their potential application in dentin formation.

## Data Availability Statement

All datasets generated for this study are included in the article/supplementary material.

## Ethics Statement

The studies involving human participants were reviewed and approved by the local ethical committee of Zhengzhou University. The patients provided their written informed consent to participate in this study.

## Author Contributions

YG, SY, and JS conceptualized and planned the experiments. YG performed all the experiments and wrote the manuscript. SY and JS contributed toward editing and proofreading the manuscript. YW and SL performed the experiments of immunofluorescence staining. WD and RG performed the experiments of quantitative polymerase chain reaction. RL was responsible for the overall project design and manuscript organization, revision, and finalization. All authors contributed to the article and approved the submitted version.

## Conflict of Interest

The authors declare that the research was conducted in the absence of any commercial or financial relationships that could be construed as a potential conflict of interest.
